# Oleic acid based experimental evolution of Bacillus megaterium yielding an enhanced P450 BM3 variant

**DOI:** 10.1186/s12896-022-00750-w

**Published:** 2022-07-13

**Authors:** Thierry Vincent, Bruno Gaillet, Alain Garnier

**Affiliations:** Department of Chemical Engineering, Université Laval, Québec, Québec G1V 0A6 Canada

**Keywords:** BM3, Experimental evolution, Biocatalysis, Enzyme engineering, p450

## Abstract

**Background:**

Unlike most other P450 cytochrome monooxygenases, CYP102A1 from *Bacillus megaterium* (BM3) is both soluble and fused to its redox partner forming a single polypeptide chain. Like other monooxygenases, it can catalyze the insertion of oxygen unto the carbon-hydrogen bond which can result in a wide variety of commercially relevant products for pharmaceutical and fine chemical industries. However, the instability of the enzyme holds back the implementation of a BM3-based biocatalytic industrial processes due to the important enzyme cost it would prompt.

**Results:**

In this work, we sought to enhance BM3’s total specific product output by using experimental evolution, an approach not yet reported to improve this enzyme. By exploiting *B. megaterium*’s own oleic acid metabolism, we pressed the evolution of a new variant of BM3, harbouring 34 new amino acid substitutions. The resulting variant, dubbed DE, increased the conversion of the substrate 10-pNCA to its product p-nitrophenolate 1.23 and 1.76-fold when using respectively NADPH or NADH as a cofactor, compared to wild type BM3.

**Conclusions:**

This new DE variant, showed increased organic cosolvent tolerance, increased product output and increased versatility in the use of either nicotinamide cofactors NADPH and NADH. Experimental evolution can be used to evolve or to create libraries of evolved BM3 variants with increased productivity and cosolvent tolerance. Such libraries could in turn be used in bioinformatics to further evolve BM3 more precisely. The experimental evolution results also supports the hypothesis which surmises that one of the roles of BM3 in *Bacillus megaterium* is to protect it from exogenous unsaturated fatty acids by breaking them down.

**Supplementary Information:**

The online version contains supplementary material available at 10.1186/s12896-022-00750-w.

## Background

In the last decades, due to their unique capacity to insert an oxygen atom into inactivated aliphatic carbon-hydrogen bonds, cytochrome P450 (CYP) enzymes have garnered much attention in the fields of biotechnology and chemistry [1–3]. Although, the oxyfunctionalization of unactivated aliphatic C–H bonds by purely chemical means exist [4–6], such synthetic pathways present major disadvantages which includes reaction conditions requiring large quantities of organic solvents [7] as well as reactions lacking in chemo-, regio-, and stereospecificity [1, 8]. This leads to much harmful waste chemicals to be disposed of in the environment and low yield reactions producing a mixture of racemic products or otherwise undesirable side products. In that respect CYP enzymes present an interesting biocatalytic alternative as they open many new potentially lucrative synthesis routes to valuable chemicals [9]. What’s more, the multiple oxidative biosynthetic and catabolic pathways in which these heme-containing enzymes are present lead to the production of a great variety of commercially relevant metabolites [10–13] with some already produced and marketed using CYP based processes [14]. The CYP which has garnered the most interest to date is that of the gram-positive bacteria *Bacillus megaterium* dubbed CYP102A1 or BM3 as it was the first CYP enzyme discovered to be both soluble and fully fused into a single polypeptide to its protein redox partners [15]. Accordingly, BM3 can be subdivided into two catalytically active domains, the oxidase domain which inserts oxygen unto inactivated aliphatic C-H bonds using atmospheric dioxygen and the reductase domain which supply electrons to the oxidase domain through its consumption of NADPH [16]. In addition, it has to date the most important catalytic activity ever reported for a CYP reaching 17 000 min^−1^ for arachidonic acid hydroxylation [17]. Yet the wild type BM3 enzyme is limited in substrates to medium or long-chain fatty acids [18] or fatty acid like compounds as substrates [19–23]. As a result, over the years, BM3 has expanded its library of oxidase active site mutants in order to broaden its range of substrates as well as its substrate chemo-, regio-, and stereospecificity for certain substrates [24]. In more recent years, the introduction of decoy molecules has further expanded the scope of substrates which could be targeted by BM3 by tricking the active site into adding oxygen unto non-native substrates [25–31]. Although much work has focused on expanding BM3 substrate range to great success, much more engineering is required to generate more stable and productive BM3 enzymes, as this has been identified as the main bottleneck preventing the industrial application of these biocatalysts. Indeed, reviews on the state of CYP biotechnology identify the operational stability of these enzymes has being the main hurdle holding back their practical implementation in biocatalytic industrial processes [32–36]. To this end, several publications have reported work focused on stabilizing point mutations to the structure of BM3. Directed evolution has been used several times to unearth stabilizing point mutations from libraries of BM3 variants generated by error prone PCR. In an early publication, the oxidase domain of BM3 harbouring the F87A mutation, which can act as a peroxygenase [37], was subjected to successive cycles of directed evolution to generate the 21B3 variant that displayed greater productivity in hydrogen peroxide driven biocatalysis than its parent, towards the substrates 12-NCA, lauric acid and styrene [38]. The 21B3 mutant would then later be further optimized, again by directed evolution, towards a more stable variant dubbed 5H6 [39]. In different work the whole BM3 sequence was targeted for directed evolution rather than just the oxidase domain where four stabilizing mutations were added to the amino acid sequence of BM3 thus generating the BM3 mutant w5f5 which was shown to be more resistant to organic solvents [40]. Interestingly it’s been described that the reductase domain of BM3 is not as stable as it’s oxidase domain [41] and, as it were, of the four mutations located on the w5f5 BM3 mutant, three were located outside of the oxidase domain [40]. Accordingly, this same disparity of domain stability would be further explored later with the aim of enhancing the overall stability of BM3 through domain swapping by removing the less stable reductase domain of CYP102A1 from *B. megaterium* to that of CYP102A3 from *Bacillus subtilis*, yielding a more stable chimera A1MA3R compared to both CYP102A1 and CYP102A3 [42]. Yet, another strategy besides fusion constructs and directed evolution that has been explored to enhance BM3’s productivity is that of consensus mutagenesis. By aligning the reductase sequence of BM3 with that of other reductase domains sharing at least 38% of amino acid sequence identity, several BM3 variants were generated by switching amino acids strongly conserved in key locations to the consensus amino acid of the aligned sequences. Some of these variants were then shown to be more stable and productive than their wild type parent [43, 44]. A similar approach was also utilized to enhance BM3’s operational productivity when CYP102A1 ecotypes had their respective domains compared in regards to their stability with some of these ecotypes displaying greater stability for either domain, thanks to but a few amino acids substitutions [45]. A technique to enhance BM3’s productivity which has not been explored as of yet is that of experimental evolution, where organisms are forced to adapt to new environmental conditions through natural selection [46, 47]. Research into BM3 revealed that the enzyme may have a role in protecting *B. megaterium* from exogenous unsaturated fatty acids by breaking them down, as it was shown that fatty acids oleic, linoleic and arachidonic acid induce BM3 expression and promote cell death [48, 49]. Correspondingly, in this work, experimental evolution was applied to *B. megaterium* to generate more productive variants of BM3 by supplying cultures of this bacterium with increasing concentrations of oleic acid starting with concentrations at which the bacteria barely survived. The mutant therein generated showed enhanced stability, productivity and activity in regard to the substrate p-nitrophenoxydecanoic acid (10-pNCA) in contrast to the wild type enzyme, despite poorer coupling efficiency in cofactor utilization for both NADPH and NADH. Additionally, several of the new amino acid added to the sequences of BM3 by this technique were strongly conserved amongst other members of the CYP102A family indicating that this technique may be utilized to more accurately target residues when using consensus mutagenesis to generate improved variants.

## Results

**Fig. 1:**
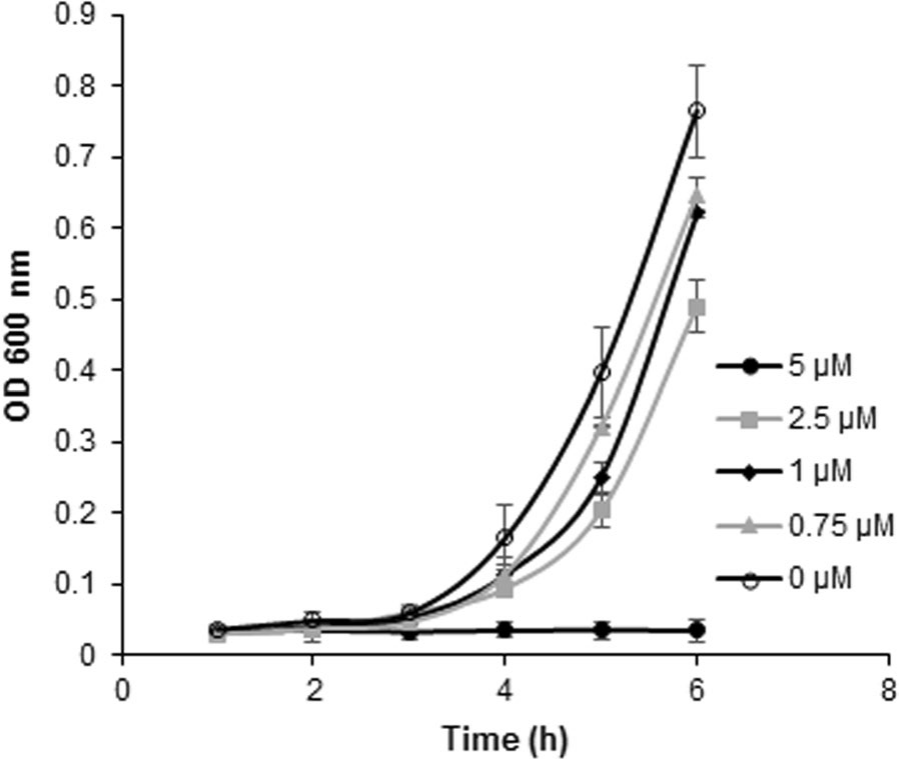
Growth kinetics of B. megaterium at various oleic acid concentrations to determine its maximum tolerable concentration in order to set up an experimental evolution experiment. Concentrations at up to 5 µM oleic acid are shown in the figure. For each data point *n* = 3, data are presented as means ± SD

**Fig. 2:**
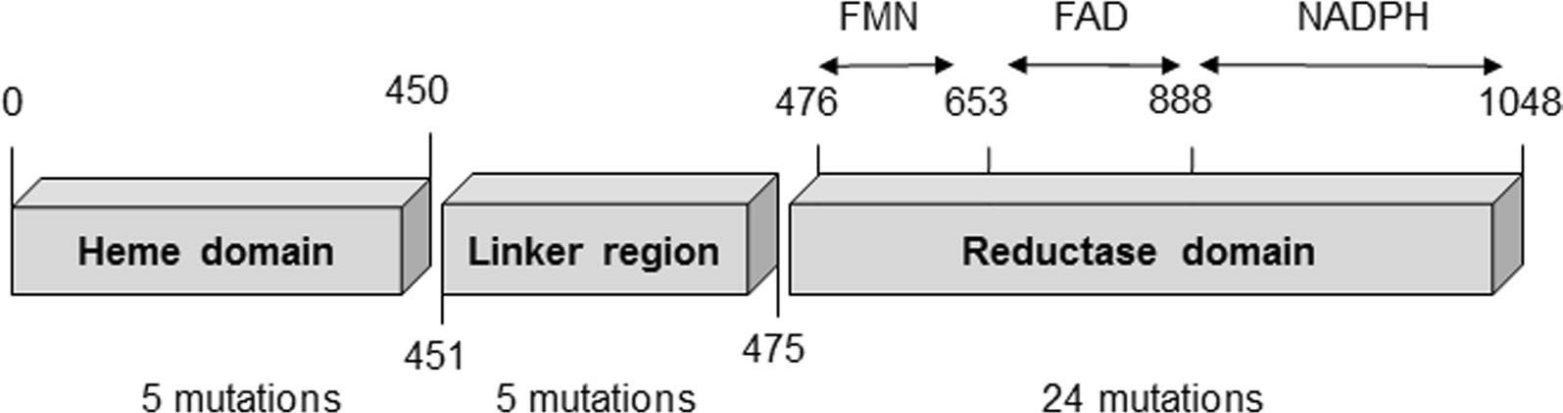
Domain architecture of the p450 BM3 cytochrome. Subdomains of the reductase domain are denoted as well above the arrows

To establish the concentration of oleic acid to be used in the experimental evolution of *B. megaterium*’s BM3 enzyme, several cultures were grown using different concentrations ranging from 0.05 to 100 µM. From these preliminary assays, the initial concentration of 2.5 µM oleic acid was selected to conduct the experimental evolution experiment, as it was the highest concentration tolerated by the bacterium, significantly slowing its growth without arresting it (Fig. 1). After several rounds of growth on both solid and liquid media at increasing oleic acid concentration up to a final concentration of 300 µM, the BM3 gene from the evolved *B. megaterium* strain was extracted and sent for sequencing. The resulting sequence accumulated over 170 nucleotide substitutions and a total of 34 mutations in the amino acid sequence compared to WT BM3. Of these 34 amino acid substitutions, 5 were located in the heme domain, another 5 were located inside the linker region and finally, the remaining 24 were all located in the reductase domain (Fig. 2, Table 1). Specifically, the flavin mononucleotide (FMN), flavin adenine dinucleotide (FAD) and NADPH subdomains of the reductase harboured respectively 6, 11 and 7 substitutions, all of which are recorded in Table 1. For simplicity, this new BM3 variant was dubbed DE. The amino acid sequence of DE was aligned against the first 9 members of the CYP102A subfamily as well as two other members of the CYP102 family, CYP102D1 and CYP102F1 (Table 2). The alignment of these sequences demonstrates that for 9 of the 34 amino acid substitutions, experimental evolution led to substitutions that followed a consensus amongst members of the same family.

**Table 1:** Amino acid substitutions accumulated by p450 BM3 DE compared to its parent sequence p450 BM3

Domains	Mutations
Heme domain	T1P, V26I, A28T, V127I & A135T
Linker region	K452Q, P463R, V470E, K473T & A474V
FMN subdomain	Q546E, L589F, D599E, V624L, D637E & K639A
FAD subdomain	G660R, T664A, Q674K, T715A, A716T, A741G, A782V, K813E, I824M, E870N & I881V
NADPH subdomain	E889G, D895G, E947K, E954N, M967V, A1008D & D1019E

**Table 2:** Sequence alignment of new evolved DE mutant compared to members of the CYP102 family

Organism	p450 Cytochrome	Mutations towards consensus									Mutations leaving consensus			
		A28T	V470E	T664A	T715A	A741G	E887G	M967V	A1008D	D1019E	Q546E	K639A	D893G	E947K
Bacillus megaterium	CYP102A1 WT	A	V	T	T	A	E	M	A	D	Q	K	D	E
	CYP102A1 DE	T	E	A	A	G	G	V	D	E	E	A	G	K
Bacillus subtilis	CYP102A2	S	Q	A	L	G	R	K	D	A	Q	K	D	D
	CYP102A3	S	A	G	L	D	G	V	D	A	G		D	E
Bacillus anthracis	CYP102A4	S	E	A	L	D	N	L	D	E	Q	K	N	T
Bacillus cereus	CYP102A5	S	E	A	L	D	N	L	D	E	Q	K	D	T
Bradyrhizobium diazoefficiens	CYP102A6	H	R	M	A	G	G	A	D	E	Q	A	D	D
Bacillus licheniformis	CYP102A7	S	G	A	F	E	G	L	A	I	E	T	D	D
Bacillus thuringiensis	CYP102A8	S	E	A	L	D	N	L	D	E	Q	K	N	T
Bacillus weihenstephanensis	CYP102A9	S	E	A	L	D	N	L	D	E	Q	K	N	T
Streptomyces avermitilis	CYP102D1	Y	Q	L	L	G	T	V	A	K	R	P	D	Q
Actinosynnema pretiosum	CYP102F1	F	V	A	V	G	S	H	A		H	R	D	E
Consensus	PUsc, S/T	E	A	Hsc, A/L	G	G	Hsc	D	E	Q	K	D	NCsc	
Secondary consensus					NCsc	Hsc								

*Pusc* Polar uncharged side chain, *Hsc* Hydrophobic side chain, *NCsc* Negatively charged side chain

**Fig. 3:**
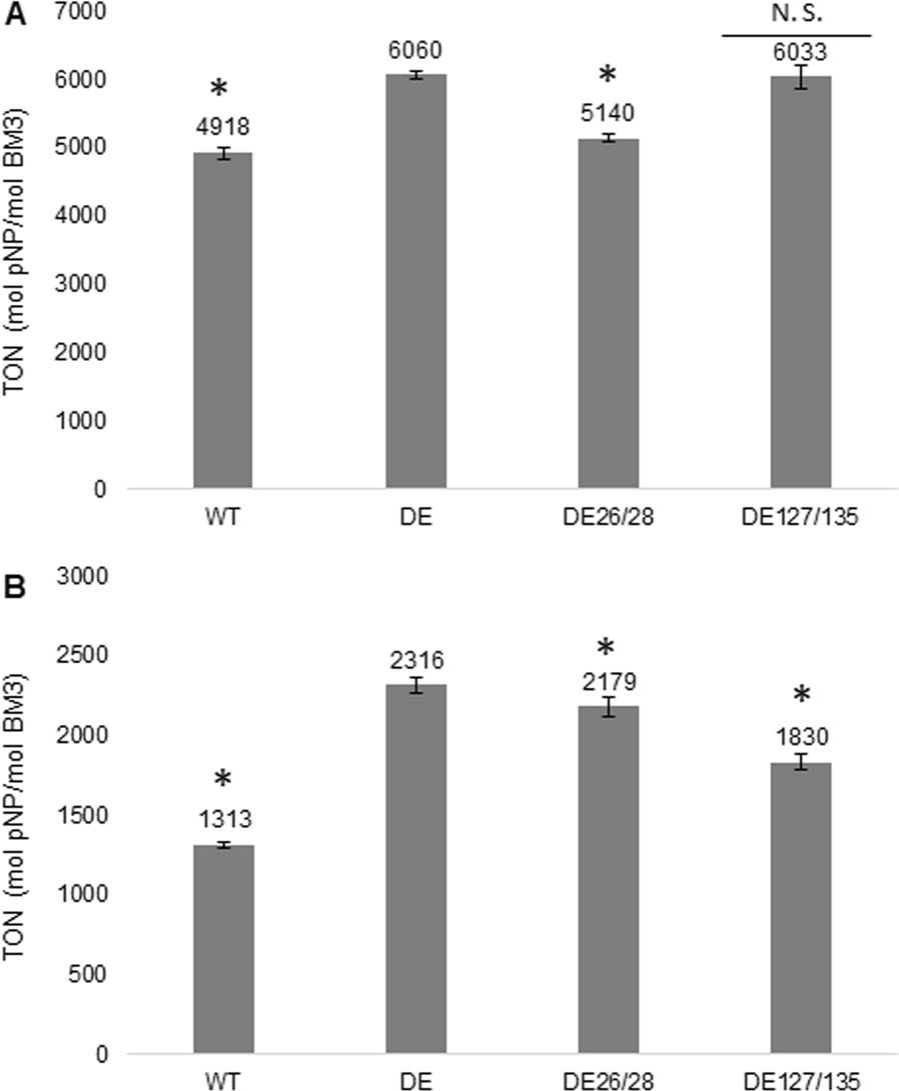
Comparison of the pNP productivity of BM3 mutant DE with mutants DE I26V/T28A, DE I127V/T135A and wild type BM3 using as cofactors either A. NADPH or B. NADH. Data are presented as means ± SD. * *P* < 0.05, N.S., not significant

**Fig. 4:**
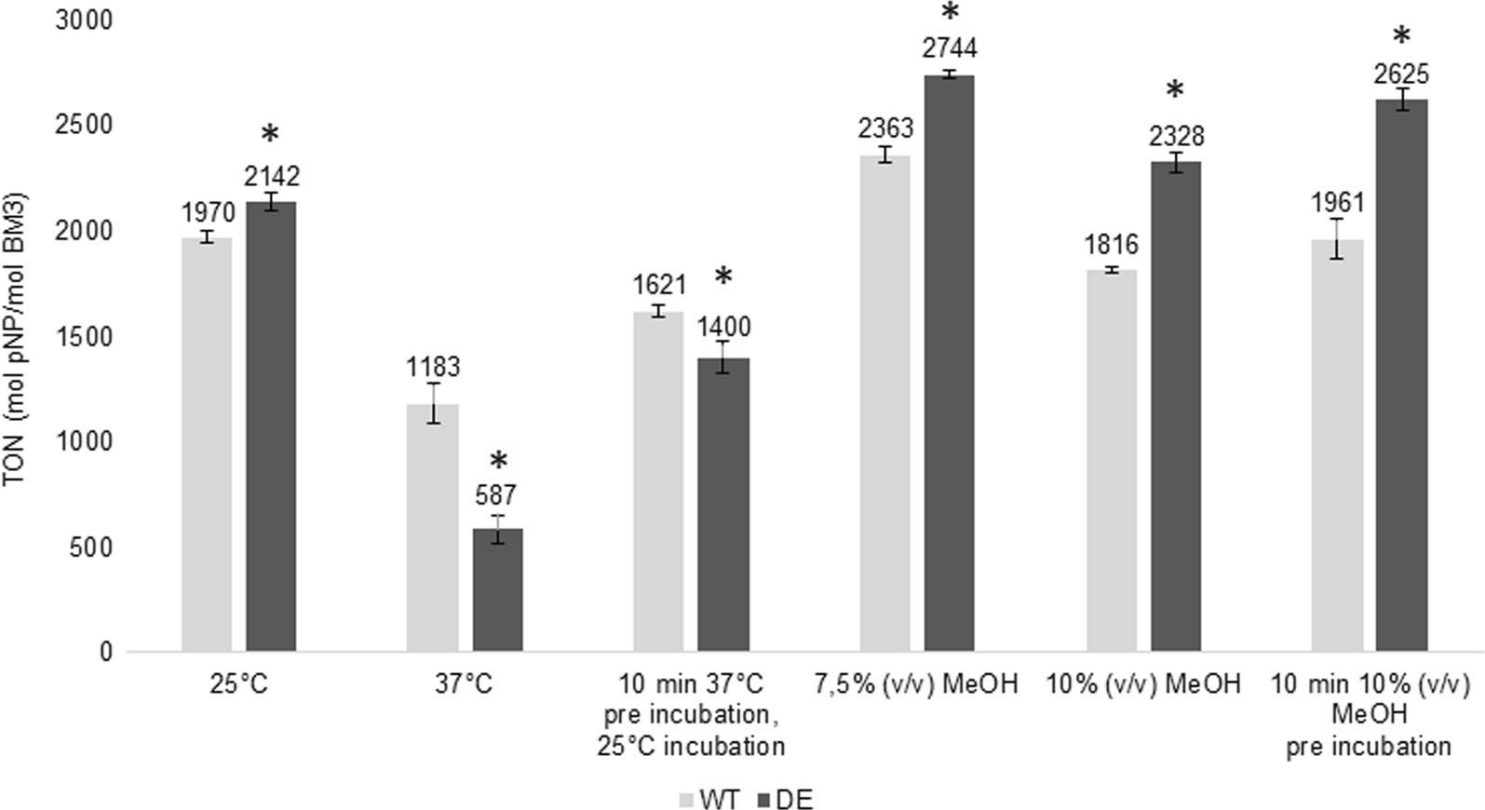
Comparison of the pNP productivity between wild type BM3 and the DE mutant in stringent conditions with thermal incubation (3 leftmost columns) or organic cosolvent concentration (methanol, 3 rightmost columns all at 4 ℃) using NADPH as a cofactor. Data are presented as means ± SD. For each condition where WT BM3 and the DE mutant is compared *P < 0.01

Following purification of the BM3 DE mutant, this new CYP was then further characterized and compared to its parent WT BM3. To assess the productivity of DE, a 10-pNCA assay was conducted with either NADPH (Fig. 3A) or NADH (Fig. 3B) as a source of cofactor. Using NADPH as a cofactor, the DE mutant proved more productive than its WT parent with a TON of 6060 pNP/CYP compared to 4918 for WT, an increase in output of 23%. Similarly, with NADH, the DE mutant proved more productive than its WT parent with a TON of 2316 pNP/CYP compared to 1313 for WT, an increase of 76%. The activity of both WT and DE BM3 were monitored with the 10-pNCA assay using NADPH, where it was determined to be of 88 and 256 min^−1^, respectively, an increase in rate of almost threefold for the mutant. In regard to the consumption rate of NADPH, WT BM3 displayed an activity of 122 min^−1^ whereas the DE variant reached 588 min^−1^. Taken together these data translate to a coupling percentage of 71.9% for WT BM3 and 43.5% for the DE variant (Table 3). A total of two new mutants were created from the DE variant where, in each case, two closely located amino acid substitutions located on the heme domain were backcrossed to their original amino acids identity to investigate their importance in DE’s performance. These two new variants dubbed DE 26/28 and DE 127/135 are thus essentially the same as the DE variant minus substitutions at amino acids 26 and 28 or 127 and 135, respectively, which were reverse-mutated to share again the same amino-acid as WT BM3. Their productivity was assessed in the presence of either NADPH or NADH as a cofactor source (Fig. 3). In the case of variant DE 26/28, compared to the DE variant, it displayed a 15.2% lower TON, 5140 pNP/CYP, and a 5.9% lower TON, 2179 pNP/CYP in the presence of either NADPH or NADH, respectively. In the case of the DE 127/135 mutant in the presence of NADPH, the observed TON was almost identical to that of the DE variant reaching 6033 pNP/CYP and not statistically different from one another. Contrastingly, in the presence of NADH the DE 127/135 mutant displayed a 21% decrease in TON compared to the DE variant, with a value of 1830 pNP/CYP. The thermal tolerance of the new BM3 variant DE was investigated and compared to that of WT BM3 by preincubating both CYPs for 10 min at 37 ℃ prior to the initiation of the reaction with NADPH and its completion at 25 °C (Fig. 4). There, WT BM3 displayed greater stability than its DE counterpart, reaching a TON of 1620 pNP/CYP whereas DE reached only 1400 pNP/CYP. Conversely, when organic solvent tolerance was assessed by preincubating both CYPs for 10 min at 4 ℃ in the presence of 10% (v/v) methanol prior to the initiation of the reaction with NADPH, the DE mutant displayed the greatest turnover reaching 2625 pNP/CYP whereas WT BM3 reached only 1961 pNP/CYP, a 34% increase in TON for the DE variant compared to WT.

**Table 3:** Kinetic data for pNP production using NADPH for WT BM3 and the DE mutant

BM3	WT	DE
*k*_*pNP*_ (min^−1^)	88 ± 13.9	256 ± 8
*k*_*NADPH*_ (min^−1^)	122 ± 53.4	588 ± 92.4
Coupling ratio (%)	71.9	43.5

k_pNP_ and k_cofactor_ are the turnover frequencies for, respectively, the production pNP from the substrate 10-pNCA in the presence of cofactors and the consumption of cofactors in the presence of 10-pNCA at the oxidase domain. Data for the catalytic constants were extracted from the slope of concentration with respect to time, obtained between *t* = 10 s and 20 s. Data are presented as means ± SD where *n* = 3

## Discussion

Much of the early work characterizing BM3 within *B. megaterium* lead us to postulate that growing this particular bacterium in the presence of a tolerable amount of any of the unsaturated fatty acids oleic, linoleic or arachidonic would apply a selective pressure on the BM3 gene to either enhance the productivity of the CYP or its expression or both. This first hypothesis was informed by the fact that the repressor protein BM3R1 which controls BM3 expression [50] can see its repression lifted when the concentration of unsaturated fatty acids increases, possibly as an adaptive response to metabolize these same fatty acids which are lethal to B. megaterium [48]. Furthermore, cultures of this bacterium, which had either been treated with nafenopin to induce BM3 expression or lacking the BM3R1 repressor altogether, displayed increased resistance against unsaturated fatty acid toxicity [49]. In an extensive review on BM3 [24], the authors surmise in regards to this information that part of the natural role of BM3 could be to detoxify such xenobiotic lipids. Interestingly, only 4% of the fatty acid content of *B. megaterium* is unsaturated, mainly 5-hexa and 5-octadecenoic acid [51]. Thus, BM3 could have other roles in the B. megaterium such as modulating membrane fluidity as branched fatty acids, all of whom are a natural substrate for BM3, comprise 80% of *B. megaterium*’s cell wall [52]. In any case, these early works informed the work accomplished here where cultures were laced with increasing concentrations of oleic acid which managed to create a variant of *B. megaterium* which could tolerate 300 µM of oleic acid, up from an initial maximum tolerable concentration of 2.5 µM.

The experimental evolution of BM3 generated a highly mutated sequence accumulating 34 mutations in total, of which only 5 were located inside the oxidase domain. In this regard Table 1 details the nature and the location of these mutations. Given the heme domain is considered to end at amino acid S450, this would make the heme domain span for 42.9% of the whole BM3 sequence. Yet, only 14.7% of mutations affected that domain leading to suppose that the oxidase domain is not the most critical region for *B. megaterium* resistance to fatty acids. The few mutations located on the heme domain prompted us to investigate whether if these were helpful at all in the performance of the new mutant, particularly V26I as it sits directly inside the heme catalytic pocket, a location far more critical to the enzyme’s activity than other positions located further away. What’s more, the A28T mutation is itself relatively close to the catalytic pocket as well. Both I127V and T135A are not as close to the catalytic pocket however even mutations far away from a catalytic pocket can have an influence on its activity through slight steric rearrangements. Given the respective proximity of V26I/A28T and I127V/T135A and the possibility for a synergistic effect on the enzyme performance both pairs were reverted to the wild type residues yielding DE26/28 and DE127/135. As denoted in our work however, these oxidase domain mutations affect TONs only slightly negatively for both DE26/28 and DE127/135 backcross mutants (Fig. 3). On the other hand, taken together, the linker region and the reductase domain span 57.1% of the whole BM3 sequence. Yet, mutations hosted within this area account for 29 of the 34 new mutations identified or 85.3% of all new mutations. This skewed distribution may arise from a need for the reductase domain to be more stable than the heme domain. Our data in regard to stability does not, however, fully support this hypothesis as, despite the new DE variant showing increased organic solvent resistance, it has less thermal tolerance than its WT BM3 parent (Fig. 4). Furthermore, the coupling ration of the DE variant is worse than that of the WT (Table 3) and thus is more likely to produce more dangerous uncoupling side products such as peroxide and superoxide anion. Interestingly, modifications to the reductase domain did come at a certain price as, in the course of this work, several other DE variants were tested with *N*-benzyl-1,4-dihydronicotinamide, a cheap alternative nicotinamide cofactor. The DE variants tested included DE W1048A, W1048S, R966D W1048S and R966D V967M W1048S. Yet, none of these DE mutants could utilize both this biomimetic cofactor and the substrate 10-pNCA, contrarily to some of the same mutations group added unto WT BM3 [44, 53].

Our initial hypothesis was that, through experimental evolution, oleic acid would enhance BM3 activity and productivity in *B. megaterium*. This was proved correct when we were able to confirm that the evolved BM3 sequence (DE) mutant displayed enhanced productivity in terms of TON (Fig. 3) and activity towards both substrate and cofactor when compared to WT (Table 3). However, the lower coupling ratio observed in DE compared to WT was unexpected, as we expected that evolution would further tame uncoupling events (Table 3). It can be assumed that for *B. megaterium* optimising the metabolic activity towards oleic acid as quickly as possible offered a greater competitive advantage over enhancing the ratios of the activities of both oxidase and reductase domains. It’s also possible that this is only the case with the substrate 10-pNCA but not with oleic acid although we did not investigate activity with this latter substrate. In any case, the objective of this work, to generate an improved scaffold of BM3 with minimal work, was successful.

An element that picked our curiosity regarding the BM3 DE amino acid sequence identity was the mechanism by which the mutated amino acids enhanced productivity and whether all of these substitutions were beneficial to the overall performance of the enzyme. In that regard, we verified whether the similarity between the new DE mutant and other members of the CYP102 family reached a measure of consensus for some of the new mutations. A total of 10 CYPs, 8 of whom belonged to the CYP102A subfamily, were selected and compared to both WT and evolved DE BM3 (CYP102A1) in regards to their amino acid sequence identity (Table 2). Noteworthy, an amino acid identity of at least 40% is required to classify CYPs within the same family and 55% within the same subfamily [54]. Of the 34 substitutions present on BM3 DE sequence, 9 replaced the WT amino acid by one similar to those found on the other members of the CYP102 family (9 leftmost columns of Table 2). This could in part explain the enhanced properties of the new sequence. This facet was not, however, further explored through mutants bearing these substitutions on the wild type sequence. Conversely, 4 mutations replaced a WT amino acid similar to the ones in other CYP102 family members with a new dissimilar one (4 rightmost columns of Table 2). This would have been another interesting feature to investigate as to whether these particular mutations helped or hindered the DE or the BM3 CYP. It is also of interest to consider that of all of the 29 amino acid substitutions located in the linker region or reductase domain, with the exception of M967V, none are located near residues identified as being potentially involved in FAD, FMN or NADPH binding [55]. In recent work [56], its been observed that there can be extensive interactions between the key residues of an active sites and more distant residues within an enzyme’s structure in effect controlling which substitutions an enzyme can tolerate. This distribution of substitutions on the reductase domain again suggests that this region of BM3 was the most critical to modify in order to enhance its resistance to oleic acid as some of these reductase mutations are strongly conserved as well as in closely related CYPs. Regardless, the sequence generated by this technique showed increased TON and activity, as was predicted when the experiment was designed. What’s more, sequences generated from this technique could serve as a powerful tool to data mine for productivity enhancing mutations to generate further improved variants.

## Conclusion

We have developed here a technique by which new improved BM3 variants can be generated with minimal work comparatively to directed evolution by error prone PCR or consensus guided evolution. In the process we have also created the BM3 DE variant, which shows increased product output of 23% and 76% compared to the WT sequence with either NADPH or NADH, respectively, as a cofactor. Once variants are sequenced and validated as being more productive, the usefulness of this technique to the engineering of more productive variants of BM3 can manifest itself in different ways once the experimental evolution is complete. For instance, the sequence could be mined for beneficial mutations that could be later added in different combinations unto the wild type BM3 sequence. Alternatively, the new sequence could be seamlessly inserted back into *B. megaterium*’s genome in place of the WT BM3 sequence and the experiment repeated further to generate a second-generation variant even further improved. Finally, yet another interesting application would be to use the technique to generate a large data set of new BM3 sequences for a machine learning approach. However, care should be taken to consider that fact that several mutations may not be beneficial to the sequence as a whole as experimental evolution does not guarantee to always produce more productive mutations. Furthermore, care should be taken as well in the use of the libraries generated by experimental evolution as opposed to directed evolution as the former generates highly mutated variants whereas the latter iteratively adds but a few mutations on each sequence every round. Thus, the benefits or drawbacks of specific mutations are easier to assess in directed evolution but harder to establish in experimental evolution. From this perspective, both techniques may also positively complement each other and contribute to the creation of more effective BM3 variants.

## Materials and methods

### Chemicals

Most chemicals used in this work were bought from either Bioshop Canada, Gold Biotechnology, Sigma Aldrich, Thermo Fisher Scientific or p212121. Else, the Pfu polymerase was purchased from Bio Basic, DNA primers were obtained from IDT technologies, *p*-nitrophenoxydecanoic acid [57] was purchased from TCI Chemicals and *N*-benzyl-1,4-dihydronicotinamide was produced by Kemitek as reported in [58]. The *B. megaterium* strain used in this work, *B. megaterium* de Bary (ATCC® 14,581™), was obtained from ATCC as was the pBR322 vector. The *Escherichia coli* dh5α and BL21 (DE3) pLysS strains were both acquired from Thermo Fischer Scientific. The restriction enzymes *SpeI*, *BamHI*, *and DpnI* were acquired from New England Biolabs. The pET-15B vector was acquired from Novagen.

### Bacillus megaterium transformation

Prior to the transformation procedure*, B. megaterium* cells were grown at 30 °C in 25 mL lysogeny broth (LB) medium (5 g/L yeast extract, 10 g/L tryptone, 5 g/L NaCl, adjusted to pH 7 with HCl) in a 250 mL shake flask at 210 rpm until cells reached an optical density at 600 nm of 1. The cells were then centrifuged at 3000 g for 10 min, resuspended into 1.5 mL fresh LB medium and then kept on ice. Following this, 475 µL of *B. megaterium* cell suspension was mixed with 15 µL of pBR322 vector DNA in 0.1 cm gapped electroporation cuvette (Bio-Rad) and incubated on ice for 30 min. The resuspendend cells were then subjected to a single electric pulse (1000 V, 50 µF, 200 Ω) using the Bio-Rad Gene Pulser Xcell^tm^. The electroporated cells were then transferred to a 1.5 mL microtube and incubated 1 h at 37 ℃ on a heating block, plated on LB agar plates containing 15 mg/mL tetracyline and incubated overnight at 30 ℃.

### Experimental evolution cultures of Bacillus megaterium

Every culture following the initial *B. megaterium* transformation contained 15 mg/mL tetracyline, were grown at 30 ℃ and stirred at 210 rpm with the exception of the 20 L bioreactor culture were cultures were stirred at 150 rpm. Plated cells were grown at 30 ℃. Unless stated otherwise, all centrifugation steps were performed at 3000 g for 10 min at room temperature. Oleic acid was dissolved in dimethyl sulfoxide (DMSO) to generate a stock solution of 158.5 µM which was used to prepare cultures containing up to 5 µM of oleic acid. A stock solution of oleic acid in DMSO of 20 mM was used to prepare cultures whose oleic acid concentration was set to be between 10 and 300 µM. These stock solutions were selected to ensure that DMSO concentration in cultures remained below 5% (v/v) so as to not interfere with bacterial growth. To establish the maximum oleic acid concentration *B. megaterium* could handle, plated transformants were first grown in a 50 mL preculture of LB medium without oleic acid in a 250 mL shake flask for 12 h. Subsequently, cells were centrifuged, resuspended in 25 mL of fresh LB medium and 500 µl of these resuspended cells were added to 50 mL of LB medium in 250 mL shake flask containing concentrations of Oleic acid ranging from 0 µM to 100 µM. These cultures were then incubated for 6 h and their optical density at 600 nm measured every hour. The standard deviation and mean for each data point were calculated from three experimental replicates.

For the experimental evolution of *B. megaterium* Additional file 1: Info 1 was added for clarity to better describe the procedure utilised for this crucial experiment. The experiment was initiated by incubating a single colony of *B. megaterium* harbouring the pBR322 vector in a 50 mL preculture of LB medium without oleic acid in a 250 mL shake flask for overnight. Cultured cells were then centrifuged, resuspended in fresh LB medium and poured inside a 20 L bioreactor containing 19.5 L of LB medium and 2.5 µM of Oleic acid. Cells were grown for 12 h at 150 rpm with pH maintained at 7 with ammonium hydroxide. These above listed tasks encompass step 1 described within Additional file 1: Info 1. At the end of the incubation, 25 mL of cultured cells were harvested and used to streak LB agar plates containing 0, 2.5 and 5 µM oleic acid for overnight incubation. Afterwards the same 25 mL was mixed with 25 mL of glycerol and stored at -80 °C in a 50 mL falcon tube. An isolated colony grown on the LB agar plates containing 5 µM of oleic acid was then grown further for 10 h in a 100 mL preculture of LB medium with 5 µM oleic acid in a 500 mL shake flask. Thereafter, 8.5 mL of the preculture was added to each of the four 2 L shake flasks containing 850 mL of LB medium with a concentration of oleic acid of either 5, 10, 20 or 30 µM. Cells were incubated for overnight. This would conclude step 2 and begin step 3 in Additional file 1: Info 1. By the end of the incubation, 25 mL of cultured cells from the shake flask with 30 µM of oleic acid were harvested and used to streak LB agar plates containing 30, 40 and 50 µM of oleic acid for overnight incubation. Afterwards the same 25 mL was mixed with 25 mL of glycerol and stored at − 80 ℃ in a 50 mL falcon tube. Following this, an isolated colony grown on LB agar plates with 50 µM of oleic acid was then further grown for 8 h in a 100 mL preculture of LB medium with 50 µM oleic acid in a 500 mL shake flask. Again, 8.5 mL of preculture was added to four 2 L shake flasks containing 850 mL of LB medium with a concentration of oleic acid of either 50, 100, 200 or 300 µM. Cells were incubated overnight. Finally, 25 mL of the 300 µM oleic acid culture was mixed with 25 mL of glycerol and stored at − 80 ℃ in a conical 50 mL tube. Another 10 mL fraction was centrifuged in a conical 15 mL tube at 8000 g for 10 min at 4 ℃ and stored at − 20 ℃ for later genomic DNA extraction.

### Genomic DNA extraction

Following experimental evolution procedures, cell pellet obtained from the centrifuged 10 mL fraction was thawed on ice while CTAB lysis buffer solution for genomic DNA extraction (2% w/v CTAB, 20 mM EDTA, 100 mM Tris–HCl, 1.4 M NaCl, pH 8) was incubated in a 65 ℃ bath to dissolve all CTAB crystals. Once ready, 400 µl of CTAB solution was used to resuspend the pellet. The mixture was then transferred to a 2 mL microtube and incubated at 65 ℃ for 30 min. Following this, 200 µl of phenol was added to the CTAB cell pellet mixture after which 200 µl of a 96/4 solution of chloroform and isoamyl alcohol was added to the mixture and gently shaked. The preparation was then centrifuged at 10,000 g for 2 min. Then, 375 µl of aqueous phase at the top was removed and transferred to a 2 mL microtube. Thereafter, 375 µl of a 96/4 solution of chloroform and isoamyl alcohol was added to the sample, gently shaked and centrifuged at 10,000 g for 2 min. Then, 350 µl of aqueous phase at the top was removed and transferred to a new 2 mL microtube to which 350 µl of isopropanol was added. The sample was then gently inverted and incubated 10 min at room temperature. Following this, the sample was centrifuged at 10,000 g for 10 min after which the supernatant was removed. In place, 750 µl of ice cold 70% ethanol was added and allowed to incubate for 2 min at room temperature. The sample was then centrifuged at 10,000 g for 5 min. Ethanol was then removed and the microtube was opened and allowed to dry. Once dry, 100 µL of milli-Q water was added to the microtube.

### Cloning, expression & purification

The new BM3 gene sequence generated by experimental evolution was synthesized by PCR from the genomic DNA extracted from the *B. megaterium* variant which had evolved to tolerate oleic acid. This genomic DNA served as a template for the forward primer 5UTR, CATTGAAAGCGGTCTGGCAAACGAGAGA, and the reverse primer 3UTR2, CATGTGAAGGTGGCGCGTGATGGA and the *pfu* polymerase to generate PCR products to send for sequencing and for cloning procedures. The PCR program for this amplification went as such: 95 ℃, 5 min / 95 ℃, 1 min / 60 ℃, 1 min / 72 ℃, 4 min, last three steps repeated 29 times followed by 72 ℃, 10 min. The WT BM3 gene sequence was similarly obtained from the genomic DNA extracted from unevolved WT *B. megaterium* 14,581. The genomic DNA therein acquired served as a template for the forward primer P450-BM3-AS2, ATGACAATTAAAGAAATGCCTCAGCC, reverse primer P450-BM3-AAS2, ACACGTCTTTTGCGTATCGG and *pfu* polymerase. The PCR program for this amplification went as such: 95 ℃, 5 min / 95 ℃, 1 min / 60 ℃, 1 min / 72 ℃, 3.5 min, last three steps repeated 29 times followed by 72 ℃, 10 min. Both evolved and WT PCR products were then further amplified using the forward primer P450-BM3-AS1, CGACTAGTATGACAATTAAAGAAATGCCTCAGCCA, and the reverse primer P450-BM3-AAS1, ATGGATCCTTACCCAGCCCACACGTCTTT. The PCR products were then subcloned into an altered pET-15B vector using *SpeI/BamHI* restriction sites. As a result, the BM3 enzyme integrated into the vector would now have an octahistidine tag (GHHHHHHHH) followed by a spacer sequence (SSGHHTSM) added to the *N*-terminus of their sequence. Backcross mutations added to the DE variant were performed by DpnI (NEB) site directed mutagenesis. The forward primer I26V/T28A, TTATTAAACACAGATAAACCGGTTCAAGCTTTGATGAAAATTGCGGATGA, and the forward primer I127V/T135A GTGCAGCTTGTTCAAAAGTGGGAGCGTCTAAATGCAGATGAGCATATT were used with the reverse primer RC1230, GTTGCTTCATGAAGAGCGAACTGCTGACCGATACACGCAC, to generate the corresponding mutations by megaprimer PCR. For mutagenesis of the DE sequence the PCR reactions were carried out with 100 ng of BM3 DE plasmid, 10 nmoles of deoxynucleotides (Bio Basic), 10 pmol of each primer and 5 units of Pfu polymerase (Bio Basic) which were then added in a 50 μL PCR reaction tube. To bring about a megaprimer, 20 cycles of PCR with an elongation time of 1.5 min/cycle were carried out followed by 30 cycles with a 20 min/cycle elongation time to allow full plasmid amplification. Afterwards, 10 units of DpnI restriction enzymes were added to the resulting PCR reactions which were then incubated at 37 ℃ for 1 h. The PCR reactions were then inserted into *E. coli* dh5a competent cells through a standard heat shock protocol and then sent for sequencing.

The production of the different recombinant BM3 enzymes was accomplished first by transforming the modified pET-15B vectors by heat shock into *E. coli* strain BL21 (DE3) pLysS on LB plates containing 100 µg/mL ampicillin. A single colony was then picked and grown overnight at 37 ℃ in 100 mL of LB medium with 100 µg/mL of ampicillin. The pre-culture medium would then be replaced by centrifuging it 10 min at 8000 g and resuspending the pellet obtained in 100 mL of fresh LB medium. An inoculum of 22,5 mL of pre-culture would then be added to 0.9 L of modified Terrific Broth (24 g/L Tryptone, 48 g/L yeast extract, 10 g/L NaCl, pH 7.6) containing 100 µg/mL of ampicillin and grown at 37 ℃. When the culture reached an OD_600_ = 0.6, induction was initiated by supplementing the culture with 1.1 mL of 1 M isopropyl β-D-1-thiogtlactopyranoside (IPTG) to a final concentration of 1 mM. In addition, 150 mL of modified Terrific Broth pH 8, 50 mL of 50% (v/v) 0.48 micron filtered glycerol and 200 μL of 100 mg/mL of ampicillin would be added to the culture, after which the induction temperature was immediately set to 28 ℃. Cells were incubated for 5–8 h and were thereafter pelleted by centrifugation at 8000 g for 10 min after which they were stored at − 80 ℃. For purification, centrifuged cells were first thawed on ice, then resuspended in 20 mL of 100 mM lysis buffer (NaCl, 25 mM Tris–HCl pH 8 with 1 mM phenylmethylsulfonyl fluoride) and lysed by sonication using 4 × 15 s bursts separated by 30 s pauses. Sonication and every following step would then be performed on ice or at 4 ℃. When sonication was complete, the suspension was clarified by centrifugation at 10,000 g, 35 min. The BM3 enzyme was purified by feeding the clarified supernatant onto 25 mL of Ni sepharose 6 fast flow resin (GE Healthcare) packed in a homemade chromatography column. The packed column was first washed with 200 mL of running buffer (40 mM NaH_2_PO_4_, 500 mM NaCl, pH 7.4). Then, the column was further washed three times with 100 mL of three different washing buffers identical in composition and pH to the running buffer but with histidine added to the solution. The first washing buffer contained 10 mM histidine, the second 20 mM histidine and the third 40 mM of histidine. The enzyme was eluted from the column with an elution buffer, again identical to the running buffer but also containing 160 mM histidine. The eluate obtained was mixed to 1 volume of glycerol yielding a 50% glycerol enzyme solution which would then be stored at − 80 ℃. In order to measure the P450 concentration, the CO difference spectroscopy method was used with an extinction coefficient of 91 mM^−1^ cm^−1^ to generate the characteristic 450 nm absorption peak of CYP enzymes [59].

### Activity assays with 10-pNCA

**Fig. 5:**
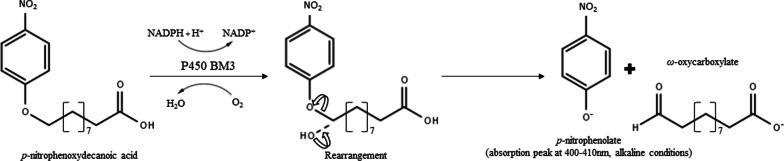
Hydroxylation of 10-pNCA catalyzed by p450 BM3

To establish the maximum turnover number (TON) the BM3 variants could generate, the spectrophotometric activity assay developed for BM3 based on the release of *p*-nitrophenolate (pNP) from p-nitrophenoxycarboxylic acids (Fig. 5)was used [60]. All enzymatic assays were performed overnight with 100 nM enzyme, 600 µM 10-pNCA and 1500 µM of either NADPH or NADH in a 100 mM Bis–Tris propane buffer with 5% (v/v) methanol, in a total volume of 1.5 mL, pH 8, 4 ℃, in darkness. Buffer solutions were always preincubated at 4 ℃ prior to the reaction to ensure the correct temperature would be maintained from the very beginning of the experiment. Noteworthy, 10-pNCA was added before the cofactor (45 µl of 20 mM 10-pNCA and 30 µl of a 75 mM cofactor stock solution). Reactions aimed at investigating thermal stability were preincubated at 37 °C for 10 min after which reactions were initiated as described above, once 10-pNCA and then NADPH was added, the samples were transferred to a 25 ℃ bath, in darkness. Likewise, the organic solvent tolerance reactions were preincubated for 10 min in a 100 mM Bis–Tris propane buffer but with 10% (v/v) methanol at 4 °C, in darkness. Reactions were initiated by the addition of 10-pNCA followed by NADPH and also performed at 4 ℃, in darkness. After an overnight incubation, 1 mL of each reaction solution was mixed with 150 µl of 10 N NaOH in a 2 mL microtube and 850 µl of methanol was added to the mixture up to a final volume of 2 mL. After this, 1 mL of this mixture was added to a 1 cm width cuvette to detect the absorbance of *p*-nitrophenolate measured at 410 nm in a Genesys 10S UV–Vis spectrophotometer (Thermo Fisher Scientific) and pNP concentration was determined from a calibration curve. To maintain pNP concentration within the range of the calibration, samples could be diluted further in methanol. For each sample set, the standard deviation and mean were calculated from 3 separate, independent experiments, each with three biological replicates. The units of TON in this work are defined as moles of pNP per mole of CYP enzyme or more simply, pNP/CYP.

Similarly, for kinetic measurements, the 100 mM Bis–Tris propane buffer, 5% (v/v) methanol, pH 8 was used at a temperature of 25 ℃. Concentrations of 100 nM (BM3) and 300 μM (10-pNCA/NADPH) were used. Reactions were performed in a final volume of 300 µl in 96 well plates. To initiate the reactions, 100 μL of 900 µM NADPH stock solution was added to 200 µl of reaction mixture containing both the enzyme and 10-pNCA. To monitor both pNP production and NADPH utilization, absorbance measurements were made at 410 nm and 340 nm, respectively using a Synergy H1 hybrid multi-mode reader (BioTek) every 5 s for 5 min with the first measure taken at *t* = 30 s. The concentrations of both pNP and NADH (or NADPH) were obtained from a calibration curve. There, the standard deviation and mean for every sample set were calculated from three experimental replicates. The units for the enzymatic activity towards the release of pNP or the consumption of NADPH are defined as moles of pNP or NADPH per moles of CYP per minute which is shortened here to min^−1^. Coupling ratio is defined as the ratio between CYP pNP production rate and NADPH consumption rate and is expressed as percentage.

## Supplementary Information

**Additional file 1: Supplementary Info 1.** Procedure for the experimental evolution of *B. megaterium* under oleic acid (Oa) selective pressure. Tet, tetracycline; OT, overnight.

## Data Availability

The datasets generated and/or analysed during the current study are available in the Figshare repository, [https://doi.org/10.6084/m9.figshare.14556195.v1].

## Abbreviations

(CYP): Cytochrome p450
(FMN): Flavin mononucleotide
(FAD): Flavin adenine dinucleotide
(LB): Lysogeny broth
(DMSO): Dimethyl sulfoxide
(pNP): *P*-Nitrophenolate
(10-pNCA): *P*-Nitrophenoxydecanoic acid
