# Supplementary figures and images for: Oleic acid based experimental evolution of Bacillus megaterium yielding an enhanced P450 BM3 variant
Source: BMC Biotechnol. 2022 Jul 13;22:20. doi: 10.1186/s12896-022-00750-w (PMC9281120; doi:10.1186/s12896-022-00750-w)

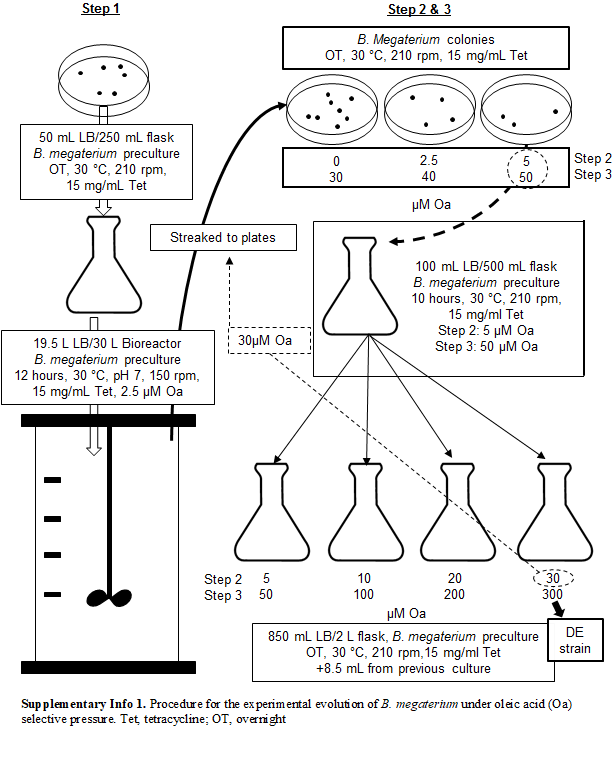

Supplement: Supplementary file 1 — Additional file 1: Supplementary Info 1. Procedure for the experimental evolution of B. megaterium under oleic acid (Oa) selective pressure. Tet, tetracycline; OT, overnight. [file 12896_2022_750_MOESM1_ESM.tif]
